# Conditional Expression of the Androgen Receptor Increases Susceptibility of Bladder Cancer in Mice

**DOI:** 10.1371/journal.pone.0148851

**Published:** 2016-02-10

**Authors:** Daniel T. Johnson, Erika Hooker, Richard Luong, Eun-Jeong Yu, Yongfeng He, Mark L. Gonzalgo, Zijie Sun

**Affiliations:** 1 Department of Urology, Stanford University School of Medicine, Stanford, CA, 94305–5328, United States of America; 2 Department of Comparative Medicine, Stanford University School of Medicine, Stanford, CA, 94305–5328, United States of America; 3 Department of Urology, University of Miami Miller School of Medicine, Miami, FL, 33136, United States of America; Florida International University, UNITED STATES

## Abstract

Bladder cancer represents a significant human tumor burden, accounting for about 7.7% and 2.4% of all cancer cases in males and females, respectively. While men have a higher risk of developing bladder cancer, women tend to present at a later stage of disease and with more aggressive tumors. Previous studies have suggested a promotional role of androgen signaling in enhancing bladder cancer development. To directly assess the role of androgens in bladder tumorigenesis, we have developed a novel transgenic mouse strain, *R26hAR*^*LoxP/+*^:*Upk3a*^*GCE/+*^, in which the human *AR* transgene is conditionally expressed in bladder urothelium. Intriguingly, both male and female *R26hAR*^*LoxP/+*^:*Upk3a*^*GCE/+*^ mice display a higher incidence of urothelial cell carcinoma (UCC) than the age and sex matched control littermates in response to the carcinogen, N-butyl-N-(4-hydroxybutyl) nitrosamine (BBN). We detect expression of the human *AR* transgene in CK5-positive and p63-positive basal cells in bladder urothelium. Further analyses of UCC tissues from *R26hAR*^*LoxP/+*^:*Upk3a*^*GCE/+*^ mice showed that the majority of tumor cells are of urothelial basal cell origin. Positive immunostaining of transgenic AR protein was observed in the majority of tumor cells of the transgenic mice, providing a link between transgenic AR expression and oncogenic transformation. We observed an increase in Ki67 positive cells within the UCC lesions of transgenic AR mice. Manipulating endogenous androgen levels by castration and androgen supplementation directly affected bladder tumor development in male and female *R26hAR*^*LoxP/+*^:*Upk3a*^*GCE/+*^ mice, respectively. Taken together, our data demonstrate for the first time that conditional activation of transgenic AR expression in bladder urothelium enhances carciongen-induced bladder tumor formation in mice. This new AR transgenic mouse line mimics certain features of human bladder cancer and can be used to study bladder tumorigenesis and for drug development.

## Introduction

Bladder cancer represents a significant human tumor burden, with more than 70,000 new cases of bladder cancer diagnosed in the nation annually, resulting in approximately 16,000 deaths [1]. It accounts for about 7.7% and 2.4% of all cancer cases in males and females, respectively [1]. However, the mortality rates of male and female patients are approximately 20.4% and 25.4%, respectively [2]. The above evidence suggests that men have a higher risk of bladder cancer, whereas women tend to have more aggressive tumors. Currently, the molecular mechanisms underlying these gender differences in bladder tumorigenesis are unclear.

Androgen signaling plays a promotional role in prostate cancer growth, and thus, androgen ablation therapy is an effective treatment for patients with naïve prostate cancer [3]. Emerging evidence has also implicated an important role of androgen signaling in bladder tumorigenesis [4–7]. Expression of the androgen receptor (AR) has been detected in both murine as well as human bladder urothelium and submucosa [7, 8]. Previous studies have suggested that androgen signaling may directly or indirectly enhance bladder cancer development [7]. Decreased bladder tumor incidence has been observed in an Ar knockout mouse model (ARKO) [5, 7]. However, it appears that there is no significant correlation between tumor grades and AR expression levels in clinical patient samples [8, 9]

A carcinogen-induced mouse model system has been frequently used to investigate the development of urothelial cell carcinoma (UCC). Mice exposed to N-butyl-N-(4-hydroxybutyl) nitrosamine (BBN) develop a spectrum of bladder pathologies, including muscle-invasive carcinomas, non-invasive carcinoma, squamous cell carcinomas, and hyperplasia [10, 11]. Interestingly, in this mouse model, a clear sexual dimorphism in bladder carcinogenesis has been observed [7, 10]. The incidence of bladder cancer in male mice was about two times greater than in female mice [7]. Moreover, neither male nor female ARKO mice developed bladder carcinoma after 12-weeks of exposure to BBN [7]. Similar to the full-body Ar knockout mice, male mice with conditional deletion of Ar in bladder urothelium by uroplakin II (UPII) promoter driven Cre were less susceptible to BBN-induced bladder carcinoma development [5]. Additionally, decreased bladder urothelium cellular proliferation was observed in both full-body and urothelial-specific Ar knockout mice [5, 7]. These studies implicate a promotional role for androgen signaling in the oncogenic transformation of bladder urothelium.

Uroplakin 3a (UPK3a) belongs to the uroplakin family, a group of integral membrane proteins [12]. The expression of Upk proteins, including Upk3a, has been observed at the luminal surface of the urothelium [13, 14]. Upk3a null mice showed the phenotypes of primary vesicoureteral reflux (VUR) and hydronephrosis [12]. *Upk3a*^*GFP/cre/ERT2/+*^ *(Upk3a*^*GCE/+*^*)* transgenic mice express an eGFPCreERT2 (Enhanced Green Fluorescent Protein and Cre-ERT2) fusion protein under the control of the mouse *uroplakin 3a* (*Upk3a*) promoter. In this mouse strain, tamoxifen-induced Cre recombinase activity has been detected in the bladder urothelium of neonatal hemizygotes [15].

To directly assess the biological role of androgen signaling in bladder tumorigenesis, we recently generated a conditional AR transgenic mouse line, *R26hAR*^*LoxP*^, in which a human *AR* transgene was specifically targeted into the ROSA26 locus, *R26hAR*^*LoxP/+*^ [16, 17]. The *AR* transgene in this mouse model can be constitutively expressed in a tissue specific manner through the activation of *Cre* recombinase [18]. We intercrossed the *R26hAR*^*LoxP/+*^ and *Upk3a*^*GCE/+*^mice in order to target inducible expression of the *AR* transgene to the bladder urothelium. Both male and female *R26hAR*^*LoxP/+*^:*Upk3a*^*GCE/+*^mice showed increased susceptibility to BBN-induced urothelial cell carcinoma (UCC) development after conditional expression of the *AR* transgene. Expression of both cytokeratin 5 (CK5) and p63, cellular markers of basal cells in the bladder urothelium, is detected in tumor cells of UCC lesions. Manipulating endogenous androgen levels by castration and androgen supplementation directly affected bladder tumor development in male and female *R26hAR*^*LoxP/+*^:*Upk3a*^*GCE/+*^ mice, respectively. Taken together, our data demonstrate for the first time that conditional activation of transgenic AR expression in bladder urothelium enhances carcinogen-induced bladder tumor formation in mice.

## Materials and Method

### Mouse Experiments

The *R26hAR-floxed* mice were generated as previously described [18]. To generate the conditional *AR* transgenic mice, we intercrossed *R26hAR*^*loxP/wt*^ mice with the *Upk3a*^*GCE/+*^ strain (JAX stock: 015855). For genotyping, mouse tail tips were isolated between age 2–3 weeks and incubated in lysis buffer (Cat# 102-T, VIAGEN Biotech, LA, CA) overnight at 55°C. Samples were briefly spun down and genomic DNA was dissolved in TE buffer. Three primers that can distinguish the wild type from the AR target allele were used for genomic PCR amplification. The forward primer, 5’-CTCTGCTGCCTCCTGGCTTCT-3’, was used for both wild type and targeted alleles, and the reverse primer for the wild type allele was 5’-CGAGGCGGATCACAAGCAATA-3’, and for targeted allele was 5’-TCAATGGGCGGGGGTCGTT-3’. PCR fragments for genotyping were amplified at 94°C for 5 min, then 94°C for 20 sec, 64°C for 30 sec, and 72°C for 25 sec for 30 cycles, then 72°C for 2 min. To assess recombination, the forward primer 5’-TTCGGCTTCTGGCGTGTGAC-3’ and the reverse primer 5’-GCTGTGATGATGCGGTAGTC-3’ were used in genomic PCR reactions. PCR fragments were amplified at 95°C for 5 min, then 95°C for 45 sec, 63°C for 50 sec, and 72°C for 2 min for 40 cycles, then 72°C for 5 min.

For castration experiments and testosterone pellet insertion, both *R26hAR*^*LoxP/+*^:*Upk3a*^*GCE/+*^ mice and their control littermates were anesthetized by IP injection with Ketamine and Xylazine. To castrate male mice, both testicles and epididymides were removed through a scrotal approach. The distal end of the spermatic cord was ligated with silk suture as described previously [19]. For pellet insertion, female mice were anesthetized as described above. The testosterone pellet was inserted subcutaneously in the back at the nape of the neck. All animal experiments performed in this study were approved by the Administrative Panel on Laboratory Animal Care at Stanford University.

### Tamoxifen Injection and BBN Treatment

Both 5-week-old *R26hAR*^*LoxP/+*^:*Upk3a*^*GCE/+*^ mice and their control littermates were administrated tamoxifen in corn oil, approximately 5 mg / 25 g body weight, by IP injection five times within a three week period. Following the last injection, at 8 weeks of age, mice were given tap water containing 0.1% of N-butyl-N-(4-hydroxybutyl) nitrosamine (BBN) ad libitum (TCI America, Portland, OR) for 12 weeks, and then normal water for one week prior to analyses. The mice were sacrificed and urinary bladders and other abnormal tissues were collected and preserved in 10% neutral buffered formalin. Mouse tissues were embedded in paraffin, sectioned, and stained with hematoxylin and eosin (H&E). At least three individual slides from each mouse were prepared and assessed for pathologic abnormalities.

### Immunohistochemistry, Immunofluorescence, and Histological Analyses

Mouse tissues were fixed in 10% neutral-buffered formalin and processed into paraffin for immunohistochemistry. Samples were cut into 5-μm sections, deparaffinized in xylene, and rehydrated using a decreasing ethanol gradient followed by PBS. Antigen retrieval was achieved by boiling tissue sections in 0.01M Citrate Buffer (pH 6.0). Tissues were then blocked with 3% hydrogen peroxide in methanol and protein blocked for 15 and 30 minutes respectively to inhibit endogenous peroxidase activity and nonspecific antibody binding, respectively. Samples were exposed to a 1:30 dilution of anti-human AR antibody (Santa Cruz Biotechnology, sc-7305), 1:300 dilution of anti-mouse/human AR (Santa Cruz, sc-816, N-20), 1:300 dilution of anti-p63 antibody (Santa Cruz, sc-8431), 1:1000 of anti Ki67 antibody (Novacastsra, NCL-ki67), 1:1000 of CK-5 antibody (Covance, PRB-160P), 1:1000 of CK8 antibody (Covance, MMS-162P), in 1% of goat serum at 4°C overnight. Slides were then incubated with biotinylated anti-rabbit or anti-mouse secondary antibody (Vector Laboratories, BA-1000 or BA-9200) for 1 hour and horseradish peroxidase streptavidin (Vector Laboratories, SA-5004) for 30 minutes at room temperature, and then visualized by DAB kit (Vector Laboratories, SK-4100). Slides were subsequently counterstained with 5% (w/v) Harris hematoxylin. For histological analysis, 5-μm serial sections were processed from xylene to water through a decreasing ethanol gradient, stained with hematoxylin and eosin, and processed back to xylene through an increasing ethanol gradient. For immunofluorescence assays, 5-μm sections were boiled in 0.01M citrate buffer (pH 6.0) for 20 minutes after re-dehydration from xylene to water, and blocked by 5% goat serum. Tissue sections were then incubated with 1:30 dilution of anti-human AR antibody (Santa Cruz Biotechnology, sc-7305), 1:300 dilution of anti-mouse/human AR (Santa Cruz, sc-816), or 1:100 dilution of anti-p63 antibody (Santa Cruz, sc-8343), 1:1000 of CK-5 antibody (Covance, PRB-160P), 1:1000 of CK8 antibody (Covance, MMS-162P), and either 1:200 of anti-GFP (Invitrogen, A11122) or 1:150 of anti-GFP (Cell Signaling, 2955) in 1% of goat serum at 4°C overnight. Goat anti-mouse Alexa Fluor 594 (Molecular probes, A21203), or goat anti-rabbit Alexa Fluor 488 (Molecular probes, A11034) was incubated at 1:500 dilution for 1 hour at room temperature. Sections were mounted by VECTASHIELD Mounting medium with DAPI (Vector Laboratories, H-1200). Preparation and examination of mTmG tissue samples was performed as previously described [20]. Images for all HE and immunohistochemistry experiments in this study were acquired on a Leica dissecting microscope (model MZ9_5_) using Zeiss Axiovision software. Immunofluorescent images were taken using an Olympus BX-52 microscope.

### Statistical Analyses

We presented the data as the mean ±SD. We made comparisons between groups using a two-sided Student’s *t* test. P<0.05 and P<0.01 were considered significant.

## Results

### Expression of Upk3a in Mouse Bladder Urothelium

**Fig 1:**
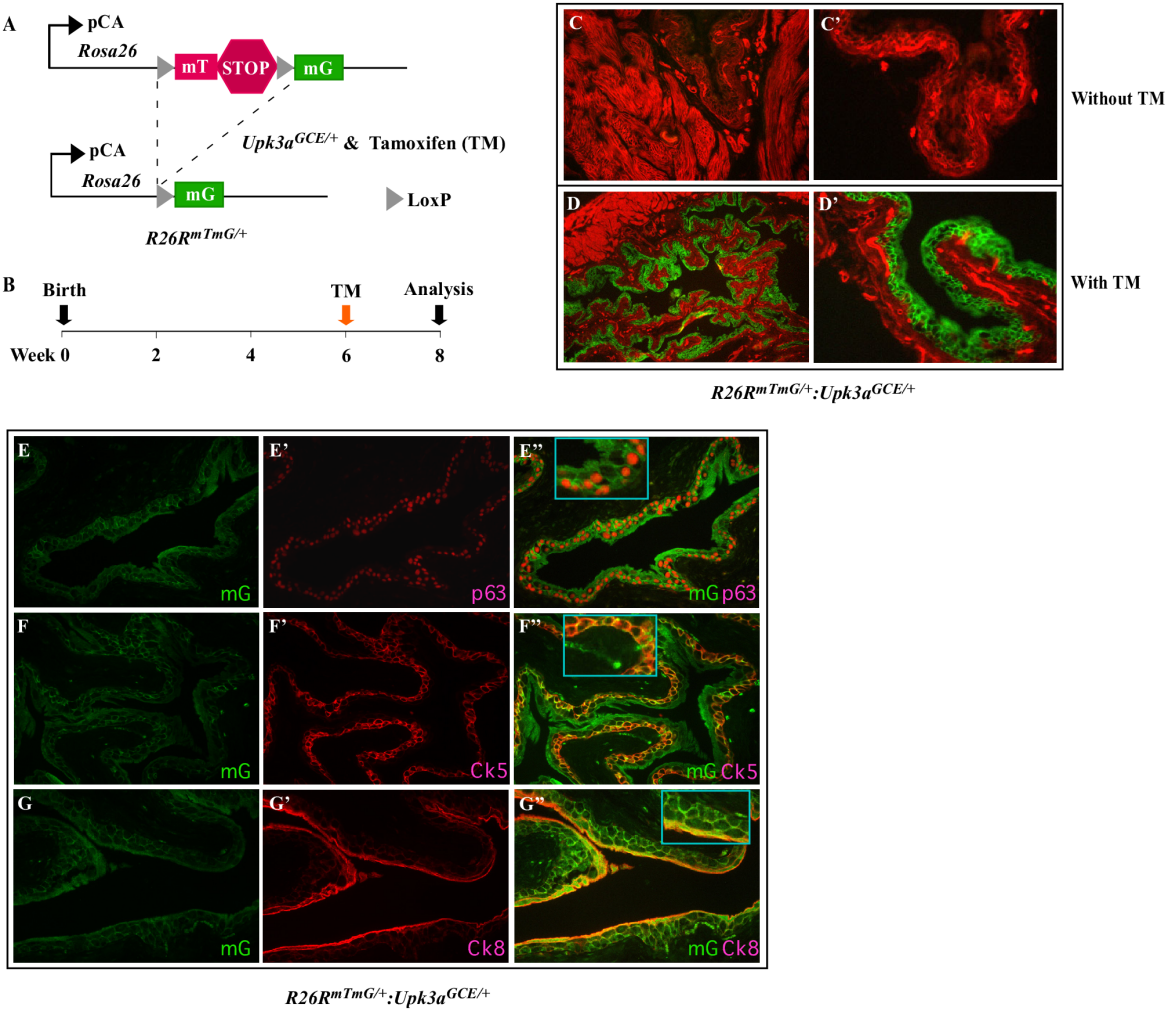
Upk3a^GCE/+^ targets Cre recombination to bladder urothelium. (**A)** Schematic demonstrating Cre-mediated labeling of Upk3a^GCE/+^-positive cells with the mT/mG reporter system. (**B)** Schedule of tamoxifen (TM) injection to induce Cre recombination of *R26*^*mTmG/+*^:*Upk3a*^*GCE/+*^ mice. (**C-C’)** Lower and higher magnification images of labeled mT, mtd-Tomato, (red) or mG, mGFP, (green) in the bladder urothelium of *R26*^*mTmG/+*^:*Upk3a*^*GCE/+*^ prior to TM injection (n = 4). (**D-D’)** Lower and higher magnification images of labeled mT (red) or mGFP (green) in the bladder urothelium in *R26*^*mTmG/+*^:*Upk3a*^*GCE/+*^ following TM injection (n = 6). (**E-G)** Immunofluorescent images of bladder urothelium of TM injected *Upk3a*^*GCE/+*^*R26*^*mTmG/+*^ mice co-stained with antibodies against GFP (E-G”) with p63 (E’-E”), CK5 (F’-F”), or CK8 (G’-G”) (n = 6).

The *Upk3a*^*GCE/+*^, transgenic mice were generated by inserting a tamoxifen-inducible Cre recombinase (CreERT2) and eGFP fusion gene under the control of the mouse *Upk3a* promoter. As reported previously, *Upk3a*^*GCE/+*^ mice appear phenotypically normal without any abnormalities [15]. To identify Upk3a expression cells, we generated *Rosa26-mT/mG* (*R26RmT/mG*):*Upk3a*^*GCE/+*^ mice by intercrossing *R26RmT/mG* [20] and *Upk3a*^*GCE/+*^ mouse strains (Fig 1A). In this mouse model, tamoxifen (TM)-induced Cre activity can switch membrane-bound tdTomato (mT) to membrane-bound green fluorescent protein (mGFP) expression through recombination of the floxed reporter loci in targeted cells. We administrated one dose of tamoxifen (TM) or vehicle control to 6-week old *R26RmT/mG*:*Upk3a*^*GCE/+*^ mice and analyzed them two weeks post injection (Fig 1B). We observed specific expression of mGFP expression in the bladder urothelium of TM-induced *R26RmT/mG*:*Upk3a*^*GCE/+*^ mice, but not in uninduced controls (Fig 1C–1D’). It has been suggested that adult rodent bladder urothelium consists of three separate populations of cells: umbrella, intermediate, and basal cells. Umbrella cells, also known as superficial cells, line the bladder lumen and highly express uroplakins and CK8, but not p63 or CK5 [10]. Intermediate cells reside underneath the umbrella cells and express both uroplakins and p63, but not CK5. Basal cells lie adjacent to the lamina propria and surrounding muscle tissues and express CK5 and p63 [21–23]. Using co-immunofluorescence assays, we further characterized the cellular identity of mGFP expression cells in the bladder urothelium with Ck5, Ck8, and p63 antibodies (Fig 1E–1G”). We observed the expression of Ck5 (Fig 1E”), p63 (Fig 1F”), and CK8 (Fig 1G”) overlaid with mGFP staining suggesting that most of the cells in bladder urothelium are targeted by *Upk3a*^*GCE/+*^ mice [21].

### Conditional Expression of the AR Transgene in Mouse Bladder Urothelium

**Fig 2:**
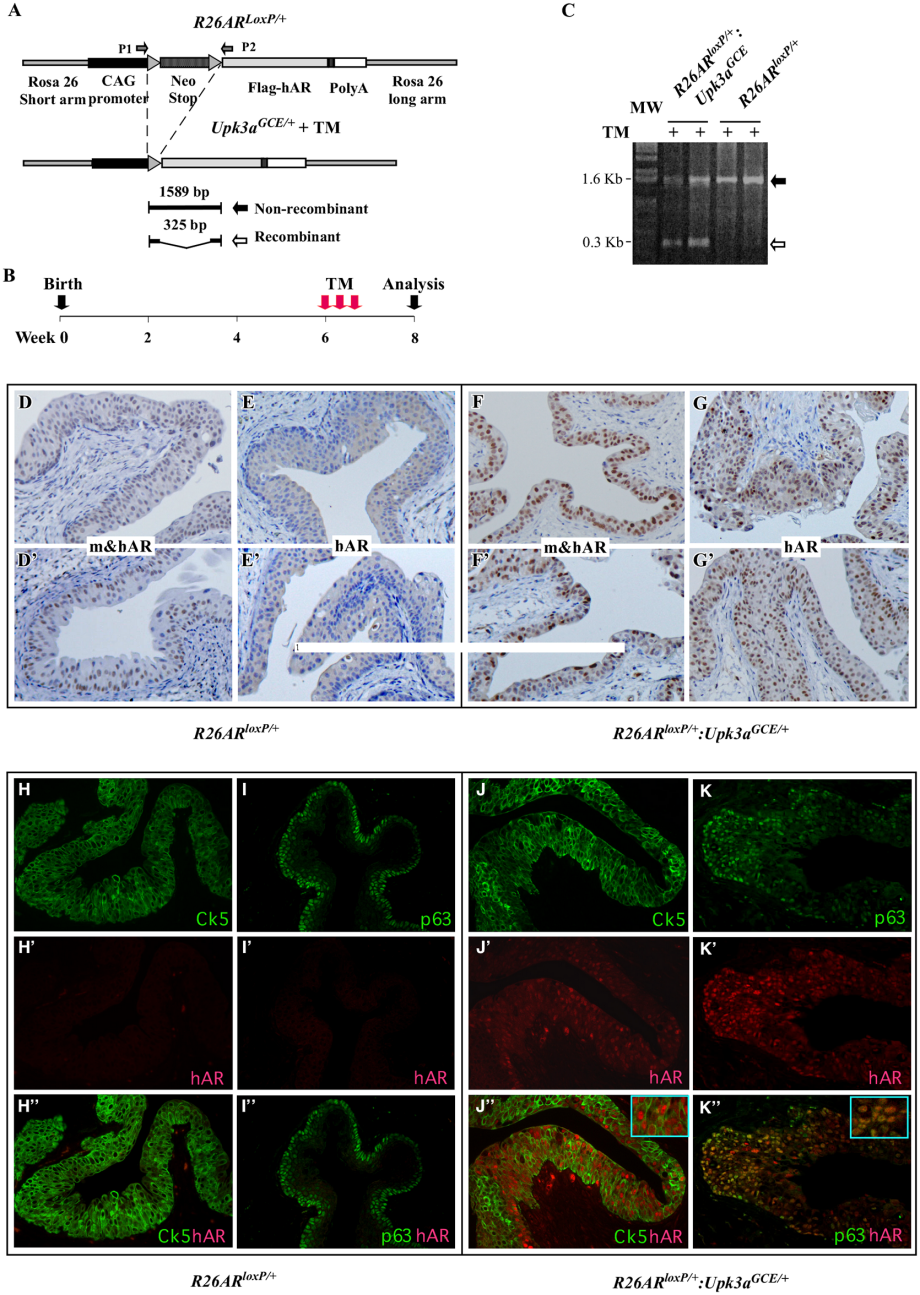
Upk3a^GCE/+^ drives conditional expression of the human *AR* transgene in mouse bladder urothelium. (**A)** Schematic depicting the Cre/LoxP-mediated recombination, resulting in deletion of the STOP cassette from the *R26AR transgene* allele. (**B)** Experimental set-up for tamoxifen-induced human *AR* transgene expression. (**C)** Genomic PCR analyses of recombination events with specific primers for the human *AR* transgene allele (see Fig 2A) with bladder urothelium tissue samples of *R26AR*^*LoxP/+*^*Upk3a*^*GCE/+*^ and *R26AR*^*LoxP/+*^ mice. The AR trangene allele was detected in the samples of both *R26AR*^*LoxP/+*^*Upk3a*^*GCE/+*^ and *R26AR*^*LoxP/+*^ mice (black arrow), but TM induced recombination on the AR transgene allele was only observed in *R26AR*^*LoxP/+*^*Upk3a*^*GCE/+*^ mice (white arrow). (**D-F’)** Immunohistochemical staining of the bladder urothelium of TM injected *R26AR*^*LoxP/+*^ mice using either the antibody that is reactive to both mouse and human AR protein (m&hAR), **or** the antibody that is reactive to only human AR (hAR) (n = 9). **(F-G’)** Immunohistochemical staining of the bladder urothelium with the two different AR antibodies as described above using the samples of TM-injected *R26AR*^*LoxP/+*^*Upk3a*^*GCE/+*^ mice (n = 12). (**H-K”)** Single channel and merged immunofluorescent images of bladder tissues of TM-injected *R26AR*^*LoxP/+*^ and *R26AR*^*LoxP/+*^*Upk3a*^*GCE/+*^ mice stained with antibodies against CK5 p63, and the human AR (in red) (n = 6).

Although the potential effect of the Ar has been assessed previously using a tissue-specific Ar knockout mouse model driven by UPII-Cre model [5], AR-mediated oncogenic transformation in bladder tumorigenesis remains unclear. To directly address the promotional role of the AR in bladder cancer initiation and progression, we generated *R26hAR*^*LoxP/+*^:*Upk3a*^*GCE/+*^ mice (Fig 2A). As demonstrated in *R26RmT/mG*:*Upk3a*^*GCE/+*^ mice, expression of transgenic AR protein can be induced in bladder urothelium after TM administration. We administered TM to *R26hAR*^*LoxP/+*^:*Upk3a*^*GCE/+*^ *and R26hAR*^*LoxP/+*^ controls starting at 5 weeks of age (Fig 2B). Using genomic PCR approaches, we confirmed the activity of *CreER* in bladder tissues, resulting in a 300 bp PCR fragment corresponding to the deletion of the *LSL* cassette through *loxP/Cre* recombination in bladder tissues of *R26hAR*^*LoxP/+*^:*Upk3a*^*GCE/+*^ mice with TM induction, but not in those of *R26hAR*^*LoxP/+*^ mice at 8 weeks of age (Fig 2C). We confirmed transgenic AR protein expression through immunohistochemistry with either an antibody (441, Santa Cruz, sc-7305) specific for transgenic human AR protein, hAR, or an antibody (N-20, Santa Cruz, sc-816) against both human and mouse AR proteins, m&hAR. Using these two different antibodies allowed us to distinguish transgenic human AR from endogenous mouse Ar in mouse bladder tissues. Nuclear AR staining with the m&hAR antibody is detected in the bladder urothelium of both *R26hAR*^*LoxP/+*^:*Upk3a*^*GCE/+*^ (Fig 2F and 2F’) and *R26hAR*^*LoxP*^ control mice (Fig 2D and 2D’), though the intensity of the former is stronger than the latter. As expected, positive nuclear staining with the human AR specific antibody, hAR, was only observed in bladder urothelial tissues isolated from *R26hAR*^*LoxP/+*^:*Upk3a*^*GCE/+*^ mice (Fig 2E and 2E’). These data indicate that expression of the *AR* transgene in bladder urothelium is a result of the *LoxP/Cre* recombination through TM-activated *Cre* transgene driven by the *Upk3a* promoter.

Using immunofluorescent approaches, we further confirmed expression of transgenic AR protein in bladder urothelium through TM-induced *LoxP/Cre* recombination in *R26hAR*^*LoxP/+*^:*Upk3a*^*GCE/+*^ mice (Fig 2J’ and 2K’), but not in *R26hAR*^LoxP/+^ only control mice (Fig 2H’ and 2I’). In line with our observation in *R26RmT/mG*:*Upk3a*^*GCE/+*^ mice, we observed a clear overlay of transgenic AR expression with both CK5 and p63 expression in urothelial cells of *R26hAR*^*LoxP/+*^:*Upk3a*^*GCE/+*^ mice (Fig 2J” and 2K”), suggesting that transgenic AR expression is present in basal and intermediate cell populations in bladder urothelium [21].

### Conditional Expression of Transgenic AR in Mouse Bladder Urothelium Enhances Susceptibility to Carcinogen-Induced Tumor Formation

**Fig 3:**
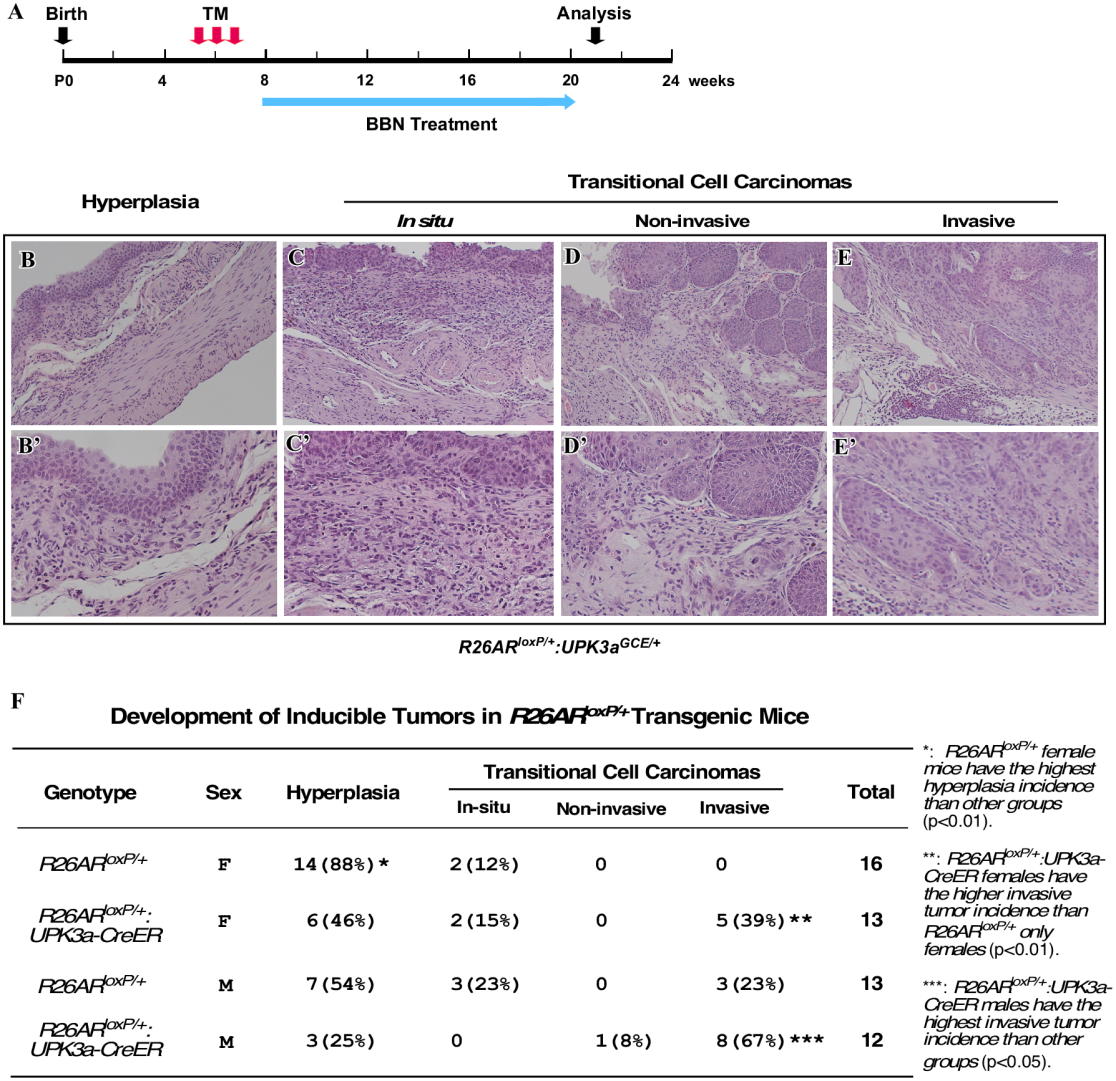
Conditional expression of the human *AR* transgene enhances BBN-induced carcinogenesis. (**A)** Experimental design to induce transgene expression and bladder carcinogenesis in *R26AR*^*LoxP/+*^ and *R26AR*^*LoxP/+*^*Upk3a*^*GCE/+*^. (**B-E)** Low (B-E) and high (B’-E’) magnification images of H&E staining depicting the range of pathological changes in *R26AR*^*LoxP/+*^*Upk3a*^*GCE/+*^ mice following TM injection and BBN treatment described in (**A)** (Hyperplasia:n = 30, Carcinoma in situ n = 5, Non-invasive carcinoma: n = 1, Invasive carcinoma: n = 17). (**F)** Analysis of the incidence of hyperplasia or urothelial cell carcinomas development in mice with different genotypes.

N-butyl-N-(4-hydroxybutyl) nitrosamine (BBN) is a well-established carcinogen that has been frequently used in rodents to induce urinary bladder cancer [24], which bears significant histopathological and molecular similarities to the human disease [25]. Therefore, we assessed whether transgenic AR expression in bladder urothelium changes the susceptibility to BBN-induced tumorigenesis. Both *R26hAR*^*LoxP/+*^:*Upk3a*^*GCE/+*^ *and R26hAR*^*LoxP/+*^ mice were administrated TM at 5 weeks of age, and were then treated with 0.1% BBN in their drinking water starting at 8 weeks of age, for 12 weeks. Mice were sacrificed 1 week after ending BBN treatment (Fig 3A). Gross and histological analyses showed a spectrum of pathological changes in bladder urothelium, which included hyperplasia, carcinoma *in situ*, non-invasive urothelial cell carcinoma, and invasive urothelial cell carcinoma (Fig 3B–3E’). Interestingly, eight out of twelve male *R26hAR*^*LoxP/+*^:*Upk3a*^*GCE/+*^ mice developed invasive urothelial cell carcinoma (67%), bearing the most severe tumor phenotypes in comparison to mice of other genotypes (Fig 3F). In contrast, only three of thirteen age matched male *R26hAR*^*LoxP/+*^ control littermates developed invasive urothelial cell carcinoma (23%). Furthermore, five out of thirteen female *R26hAR*^*LoxP/+*^:*Upk3a*^*GCE/+*^ mice also developed invasive urothelial cell carcinomas (39%) while no age-matched female *R26hAR*^*LoxP/+*^ control littermates developed either non-invasive or invasive urothelial cell carcinomas (Fig 3F). These data demonstrate that conditional expression of transgenic AR in bladder urothelium cells enhances susceptibility to oncogenic transformation and tumor aggressiveness in both male and female *R26hAR*^*LoxP/+*^:*Upk3a*^*GCE/+*^ mice with BBN induction.

### BBN-Induced Urothelial Cell Carcinomas in *R26hAR*^*LoxP/+*^:*Upk3a*^*GCE/+*^ Are Urothelial Intermediate and Basal Cell Origin

**Fig 4:**
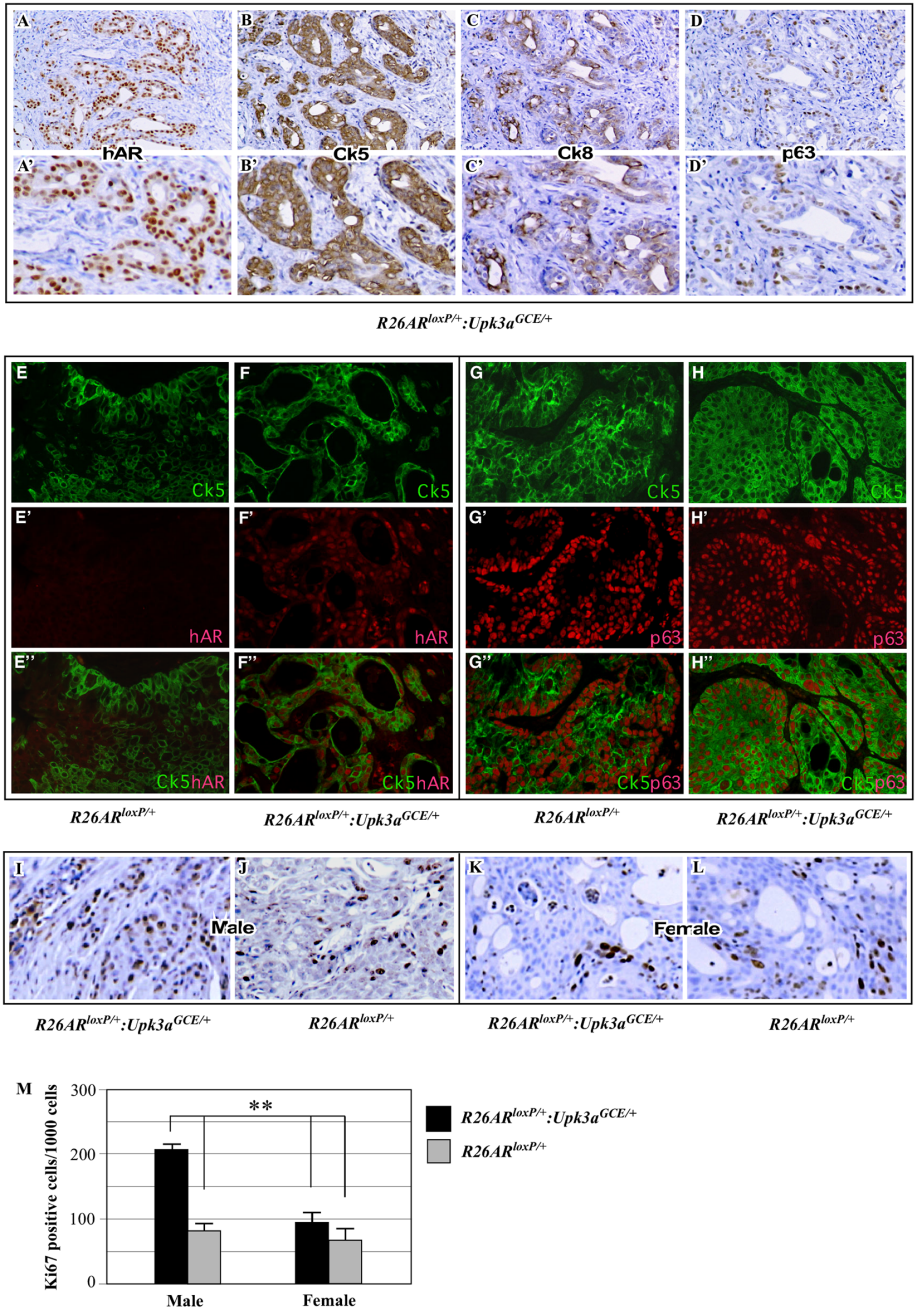
Analysis of cellular identity of bladder tumors following conditional AR expression and BBN-treatment. (**A-D)** Low (A-D) and high (A’-D’) magnification images of immunohistochemical staining with the antibody against the human AR, hAR (A-A’) and CK5 (B-B’), CK8 (C-C’), or p63 (D-D’) in bladder tumors from *R26AR*^*LoxP/+*^*Upk3a*^*GCE/+*^mice (n = 3). (**E-F”)** Immunofluorescent analyses of bladder tumor tissues isolated from *R26AR*^*LoxP/+*^ and *R26AR*^*LoxP/+*^*Upk3a*^*GCE/+*^mice. Single channel and merged images showing CK5 (green) and human AR (red) co-localization in bladder tumors in mouse tissues (n = 3). (**G-H”)** Similar analyses as described above were performed using the antibody against CK5 (green) or p63 (red) in bladder tumors from *R26AR*^*LoxP/+*^ and *R26AR*^*LoxP/+*^*Upk3a*^*GCE/+*^mice (n = 4). (**I-L)** Immunohistochemical analyses of bladder tumor tissues isolated from male or female *R26AR*^*LoxP/+*^ and *R26AR*^*LoxP/+*^*Upk3a*^*GCE/+*^mice using an antibody against the proliferation marker, Ki67 (n = 3). (**M)** Quantification of Ki67-positive cells detected in carcinomas from both genotypes in male and female mice (n = 3).

It has been shown that BBN induced the development of bladder urothelial cell carcinoma in mice [6, 22]. To further define the origin of bladder tumors in the *R26hAR*^*LoxP/+*^:*Upk3a*^*GCE/+*^ mice, we performed immunohistochemical analyses to examine a series of cellular markers in tumor tissues. As shown in Fig 4A–4D’, tumor cells showed positive immunostaining with the human AR antibody (Fig 4A and 4A’), suggesting a link between transgenic AR expression and BBN-induced oncogenic transformation. Tumor cells showed strong positive staining for CK5 (Fig 4B and 4B’), which is a hallmark of pathologies arising from the basal cells [22]. Slight staining of CK8 was also observed in tumor cells (Fig 4C and 4C’). Most tumor cells were also reactive to p63 antibody (Fig 4D and 4D’). Using co-immunofluorescent approaches, we further characterized the cellular identity of tumor cells. Nuclear staining of transgenic AR overlaid uniformly with cytoplasmic membrane staining of CK5 in tumor cells of *R26hAR*^*LoxP/+*^:*Upk3a*^*GCE/+*^ mice (Fig 4F–4F”). Additionally, most CK5 positive tumor cells isolated from both *R26hAR*^*LoxP/+*^:*Upk3a*^*GCE/+*^ and *R26hAR*^*LoxP/+*^ mice were also reactive to p63 antibodies (Fig 4G–4H”). Taken together, these data suggest that the origin of BBN-induced bladder urothelial cell carcinoma is mainly derived from basal cells in *R26hAR*^*LoxP/+*^:*Upk3a*^*GCE/+*^ mice and *R26hAR*^*LoxP/+*^ controls.

### Transgenic AR Expression Promotes Tumor Cell Proliferation in the Bladder of *R26hAR*^*LoxP/+*^:*Upk3a*^*GCE/+*^ Mice

A promotional role of AR in cellular proliferation has been previously demonstrated in the prostate [26–28]. We investigated the potential effect of transgenic AR expression in promoting cellular proliferation in bladders of BBN-treated *R26hAR*^*LoxP/+*^:*Upk3a*^*GCE/+*^ mice. We performed immunohistochemistry to stain bladder tissue slides with Ki67 antibody from both male and female R*26hAR*^*LoxP/+*^:*Upk3a*^*GCE/+*^ and R*26hAR*^*LoxP/+*^ mice (Fig 4I–4L). Ki67 immunostaining was quantified by counting a total of 1000 bladder urothelial cells from five high-power fields in each sample. Three mice in each sex and genotype were analyzed, representative data is shown (Fig 4I–4L). The proliferative index in the male *R26hAR*^*LoxP/+*^:*Upk3a*^*GCE/+*^ group was approximately one-fold greater than in male *R26hAR*^*LoxP/+*^ control mice, and female *R26hAR*^*LoxP/+*^ and *R26hAR*^*LoxP/+*^:*Upk3a*^*GCE/+*^ mice (Fig 4M). These results implicate a promotional role of transgenic *AR* expression in cellular proliferation of BBN-induced bladder urothelial tumors.

### Androgens Play a Dominant Role in Increasing the Susceptibility to BBN Induced Urothelial Carcinoma Development in *R26hAR*^*LoxP/+*^:*Upk3a*^*GCE/+*^ Mice

**Fig 5:**
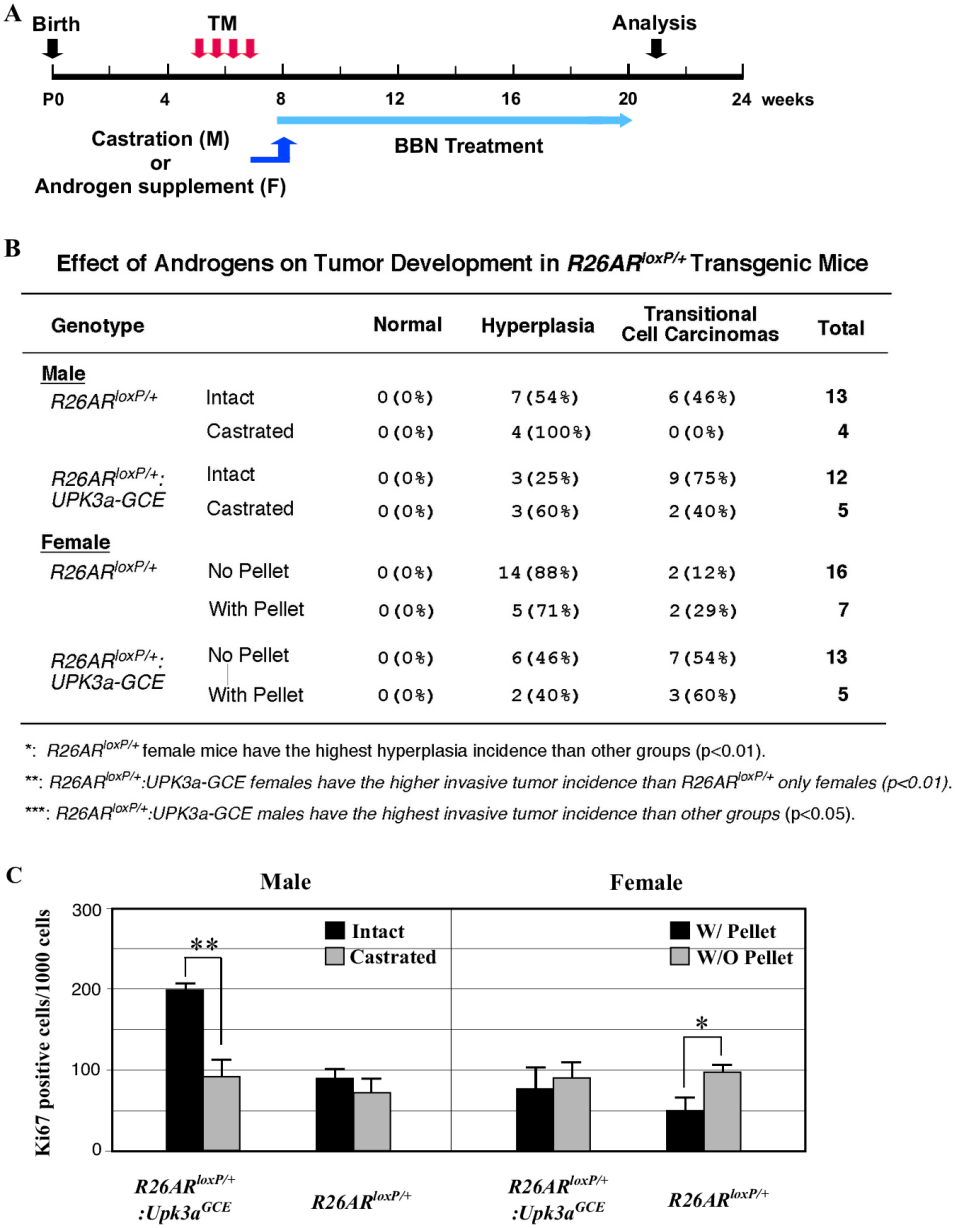
The effect of androgens on tumor development in *R26AR*^*LoxP/+*^ and *R26AR*^*LoxP/+*^*Upk3a*^*GCE/+*^mice. (**A)** Schematic depicting experimental design for castration of male mice and testosterone pellet insertion of female mice. (**B)** Analysis of the androgen effect on different genotypes of male and female mice with castration and testosterone pellet insertion. (**C)** Quantification of Ki67-positive cells in castrated and intact male mice and female mice with and without testosterone pellet (n = 4).

In this study, we observed the highest incidence of BBN-induced invasive urothelial cell carcinomas in male *R26hAR*^*LoxP/+*^:*Upk3a*^*GCE/+*^ mice compared to other sex and genotype mice. In addition, tumor cells from *R26hAR*^*LoxP/+*^:*Upk3a*^*GCE/+*^ mice appeared to be more proliferative than those from other groups of mice. The AR is a member of the steroid hormone receptor superfamily [29], and its activity is regulated through androgens in general. To further characterize the role of the androgen axis in *R26hAR*^*LoxP/+*^:*Upk3a*^*GCE/+*^ mice, we manipulated androgen levels by either castrating male mice or supplementing female mice with androgen pellets. We then assessed BBN-induced bladder tumorigenesis in the above mice (Fig 5A). Intriguingly, castrated male *R26hAR*^*LoxP/+*^ control mice only developed bladder urothelial hyperplasia (Fig 5B). However, 2 out of 5 (40%) castrated *R26hAR*^*LoxP/+*^:*Upk3a*^*GCE/+*^ mice developed urothelial carcinomas. As we previously observed, intact male *R26hAR*^*LoxP/+*^:*Upk3a*^*GCE/+*^ mice showed the highest incidence of urothelial cell carcinomas among the various groups of mice in this study (Fig 5B).

To assess the role of androgen signaling during BBN-induced bladder tumorigenesis in female mice, we implanted androgen pellets and then administrated BBN to female *R26hAR*^*LoxP/+*^:*Upk3a*^*GCE/+*^ and *R26hAR*^*LoxP/+*^ mice (Fig 5A). Two out of sixteen (12.5%) female *R26hAR*^*LoxP/+*^ control mice without androgen supplementation developed bladder urothelial carcinomas (Fig 5B). However, 7 out of 13 (54%) female *R26hAR*^*LoxP/+*^:*Upk3a*^*GCE/+*^ without androgen supplementation developed urothelial carcinomas. With androgen supplementation, females with *R26hAR*^*LoxP/+*^:*Upk3a*^*GCE/+*^ genotype have a higher incidence of urothelial cell carcinomas (60%) compared to *R26hAR*^*LoxP/+*^ controls (29%). Taken together, these data provide additional line of evidence demonstrating that the promotional effect of transgenic AR protein in BBN-induced bladder tumorigenesis is mediated through androgens.

In this set of experiments, we examined cellular proliferation using Ki67 immunohistochemistry with samples isolated from different genotype, treatment, and gender groups. As shown in Fig 5C, we observed higher Ki67 staining from samples isolated from intact *R26hAR*^*LoxP/+*^:*Upk3a*^*GCE/+*^ mice than those from castrated counterparts. There was no significant difference in Ki67 staining between intact and castrated *R26hAR*^*LoxP/+*^ male mice, as well as female *R26hAR*^*LoxP/+*^:*Upk3a*^*GCE/+*^mice that either received or did not receive androgen pellet insertion. Interestingly, tumor cells in the female *R26hAR*^*LoxP/+*^ mice without pellet supplement showed higher Ki67 staining than tumor cells from the mice with pellets (Fig 5C), which implies that other signaling pathways may also be involved in regulating tumor cell proliferation in BBN-induced bladder urothelial tumors in this group of mice.

## Discussion

In this study, we directly address a long-unanswered question regarding the biological role of androgen signaling in bladder tumorigenesis. Using *R26RmT/mG*:*Upk3a*^*GCE/+*^ reporter mouse line, we detected the activity of the Upk3a promoter in the bladder urothelium of mice. We then generated the *R26hAR*^*LoxP/+*^:*Upk3a*^*GCE/+*^ mouse strain to directly assess the role of conditional expression of the AR in the basal and intermediate cells in bladder urothelium by BBN-induced bladder tumorigenesis. The data generated from this study provide the first line of evidence that demonstrates a promotional role of transgenic AR expression in enhancing the susceptibility to BBN-induced bladder urothelial carcinomas in mice.

Sexual dimorphism has been observed among human bladder cancer patients. Therefore, a potential role for androgen signaling as a contributing factor to the pathogenesis of bladder cancer has long been speculated. Initial studies examining AR expression in tissue lysates prepared from bladder tumor biopsies detected high levels of AR expression in both male and female patients when compared to samples from normal bladder urothelium [30]. Similar observations were also reported in bladder patient samples by using immunohistochemical approaches [8, 9, 31]. Earlier studies also showed decreased bladder tumor incidence in the Ar knockout mouse models, suggesting a protective role of AR deletion [5, 7]. However, previous studies have not directly assessed the promotional role of the AR in bladder tumorigenesis using biologically relevant and appropriate *in vivo* systems. In this study, we addressed the biological significance and consequences of transgenic AR expression in bladder urothelial tumorigenesis using a new and unique mouse model, *R26hAR*^*LoxP/+*^:*Upk3a*^*GCE/+*^mice. We observed that conditional expression of the AR in bladder urothelium significantly enhances BBN-induced bladder cancer development in both male and female mice. These data suggest that dysregulation of AR activation may directly contribute to the sexual dimorphism observed in human bladder cancer development. In addition, our *R26hAR*^*LoxP/+*^:*Upk3a*^*GCE/+*^ mouse model appears to be a unique and innovative tool that can be used to characterize the molecular mechanisms underlying AR action in bladder tumorigenesis.

Given the fact that the AR is a steroid hormone receptor and its activation is mainly through androgen signaling, we carefully characterized the effect of androgen axis in *R26hAR*^*LoxP/+*^:*Upk3a*^*GCE/+*^ mice. Decreasing androgen levels by castration showed an inhibitory effect of BBN-induced tumor progression from bladder urothelial hyperplasia to urothelial carcinomas in both male *R26hAR*^*LoxP/+*^:*Upk3a*^*GCE/+*^ mice and age- and sex-matched *R26hAR*^*LoxP/+*^ control littermates. Moreover, conditional expression of transgenic AR in female *R26hAR*^*LoxP/+*^:*Upk3a*^*GCE/+*^ mice increased the incidence of BBN-induced bladder urothelial carcinomas compared to female *R26hAR*^*LoxP/+*^ control littermates. Keeping consistent to this line of evidence, androgen-supplemented female *R26hAR*^*LoxP/+*^:*Upk3a*^*GCE/+*^ mice demonstrated the highest incidence of BBN-induced urothelial carcinoma compared to both untreated counterparts and female *R26hAR*^*LoxP/+*^ control littermates. Our data from this series of experiments suggest that androgen signaling enhances the susceptibility to BBN-induced bladder tumorigenesis in both genders.

The AR is a ligand-dependent transcription factor that upon binding to androgens can translocate to the nucleus, bind to androgen-responsive elements in DNA and activate transcription [32]. Transcriptional downstream target genes of the AR have been well investigated, particularly in male reproductive organs including the prostate. However, the downstream targets of AR in the bladder have not been well characterized. Interestingly, recent studies have shown increased Wnt/β-catenin signaling in response to AR activation in several bladder cancer cell lines [33, 34]. In a mouse model expressing stabilized β-catenin, a three-fold decrease in bladder tumor incident was observed in castrated male mice in comparison to intact counterparts, implicating a link between the oncogenic effects of Wnt/β-catenin signaling and the androgen axis [34]. In this study, we examined the expression and cellular localization of β-catenin in bladder tissues of *R26hAR*^*LoxP/+*^:*Upk3a*^*GCE/+*^ and *R26hAR*^*LoxP/+*^ mice. However, we did not observe any significant difference in β-catenin expression and cellular distribution between urothelial carcinoma cells in different mice genotypes (S1 Fig). We also examined the activity of Wnt/β-catenin signaling by examining the levels of the β-catenin downstream target, c-Myc, but also did not observe any significant differences between the tumor samples isolated from AR transgenic mice and controls (S1 Fig). It has also been suggested that CD24 was a downstream target of androgen signaling in bladder tissues, and may play a role in bladder tumorigenesis [35]. In the same study, it has been shown that deletion of CD24 reduced BBN-induced bladder carcinogenesis, and that tumor reduction effect was more striking in male than in female mice [35]. CD24 expression and related activity have also been correlated with decreased tumor growth and metastasis in both xenograft and transgenic knockout mouse models of bladder cancer [35, 36]. However, we failed to detect CD24 expression in bladder tissue samples isolated from both *R26hAR*^*LoxP/+*^:*Upk3a*^*GCE/+*^ and *R26hAR*^*LoxP/+*^ mice by using a variety of experimental approaches (data not shown). Currently, we do not know the reasons for the discrepancies between our results and previous reports. Given that the molecular mechanisms for controlling CD24 expression and activity remain elusive, more in-depth experiments using our current new mouse model are necessary to determine the regulatory role of androgen signaling in CD24 expression.

The pioneering work that depletion of androgens resulted in significant tumor regression in prostate cancer patients by Charles Huggins and Clarence Hodges demonstrated a key role of androgen-signaling in human prostate cancer [3]. The promoting role of androgen signaling through the AR in stimulating prostatic tumor cell growth has been well studied in human prostate cancer. However, it is still unclear whether androgen signaling can enhance or promote oncogenic transformation during the course of tumor initiation and progression in other tissues and organs. In this study, we used the BBN-induced urinary bladder carcinoma model to assess the potential role of androgen signaling in bladder tumorigenesis. We demonstrate a promotional role of androgen signaling in enhancing the susceptibility of BBN-induced bladder tumorigenesis. Using different experimental approaches to manipulate endogenous androgen levels, we have shown that the effect of transgenic AR expression in bladder urothelial cells is mediated through androgens in both male and female mice. Moreover, we observed that transgenic AR expression increases cellular proliferation. Given the nature of our study using the BBN-induced bladder tumor model, our findings also suggest that other factors may play a dominant role in inducing bladder tumor initiation and progression. Therefore, future studies using *R26hAR*^*LoxP/+*^:*Upk3a*^*GCE/+*^ mice combined with other relevant mouse models may explore the collaborative effects between androgen signaling and other signaling pathways in bladder tumor initiation and progression.

## Supporting Information

S1 Fig Detection of Wnt/β-catenin signaling in the AR transgenic mice. (JPG)
