# Supplementary figures and images for: Conditional Expression of the Androgen Receptor Increases Susceptibility of Bladder Cancer in Mice
Source: PLoS One. 2016 Feb 10;11(2):e0148851. doi: 10.1371/journal.pone.0148851 (PMC4749068; doi:10.1371/journal.pone.0148851)

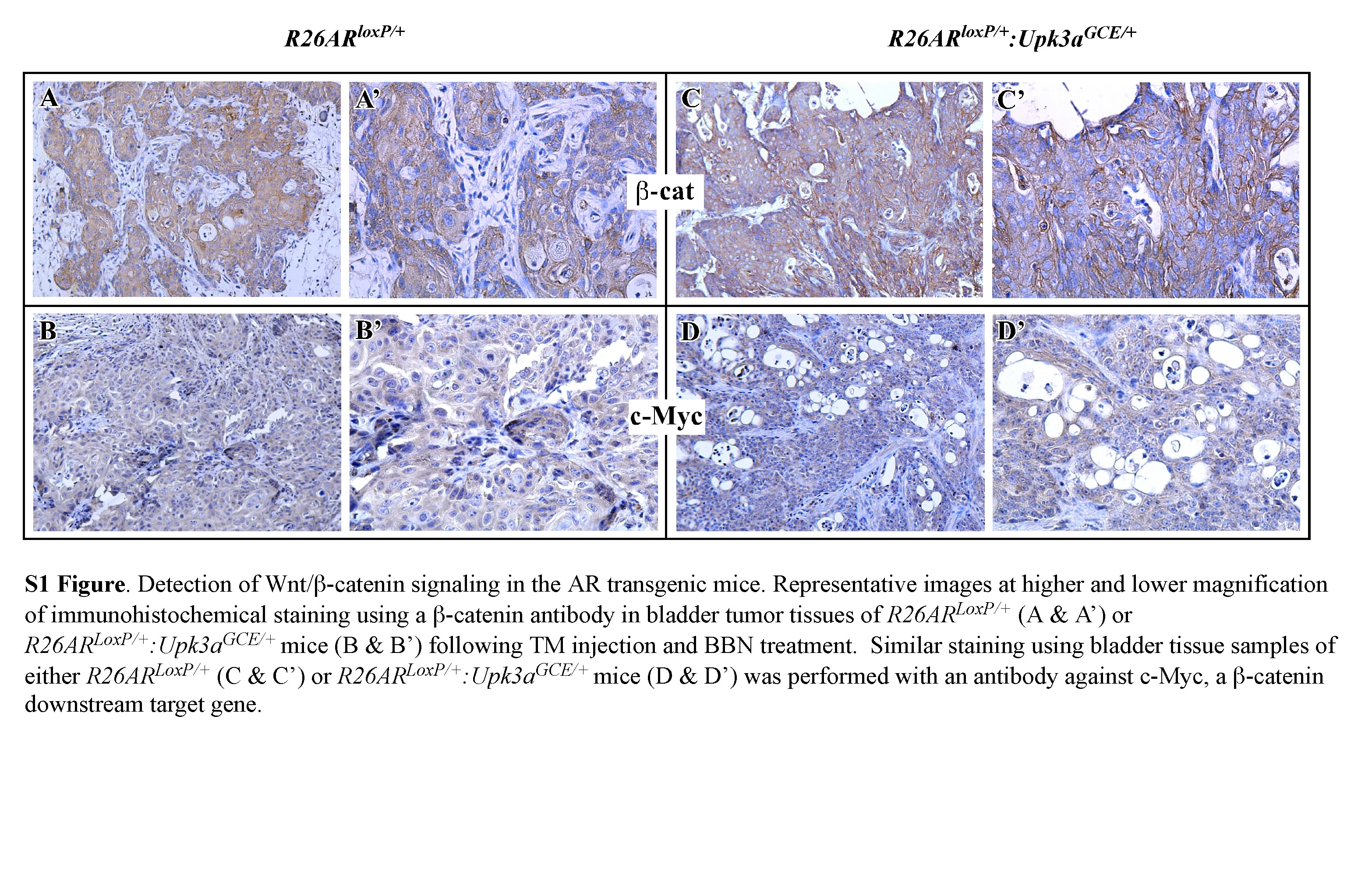

Supplement: S1 Fig — (JPG) [file pone.0148851.s001.jpg]
